# Intravital assessment of myelin molecular order with polarimetric multiphoton microscopy

**DOI:** 10.1038/srep31685

**Published:** 2016-08-19

**Authors:** Raphaël Turcotte, Danette J. Rutledge, Erik Bélanger, Dorothy Dill, Wendy B. Macklin, Daniel C. Côté

**Affiliations:** 1 Centre de recherche de l’Institut Universitaire en Santé Mentale de Québec, Université Laval, Québec, QC G1J 2G3, Canada; 2 Department of Cell and Developmental Biology, School of Medicine, University of Colorado Anschutz Medical Campus, Aurora, CO 80045, USA; 3 Centre d’Optique, Photonique et Laser, Université Laval, Québec, QC G1V 0A6, Canada

## Abstract

Myelin plays an essential role in the nervous system and its disruption in diseases such as multiple sclerosis may lead to neuronal death, thus causing irreversible functional impairments. Understanding myelin biology is therefore of fundamental and clinical importance, but no tools currently exist to describe the fine spatial organization of myelin sheaths *in vivo*. Here we demonstrate intravital quantification of the myelin molecular structure using a microscopy method based on polarization-resolved coherent Raman scattering. Developmental myelination was imaged noninvasively in live zebrafish. Longitudinal imaging of individual axons revealed changes in myelin organization beyond the diffraction limit. Applied to promyelination drug screening, the method uniquely enabled the identification of focal myelin regions with differential architectures. These observations indicate that the study of myelin biology and the identification of therapeutic compounds will largely benefit from a method to quantify the myelin molecular organization *in vivo*.

The myelin sheath surrounding axons provides functional and structural support to neuronal communication in both the central and peripheral nervous systems^1^. Its loss in a multitude of human demyelinating diseases (multiple sclerosis, Guillain-Barré syndrome, neuromyelitis optica, etc.) can result in neurodegeneration and therefore severely affect patients’ health^2^,^3^,^4^. Other myelin disorders are characterized by an abnormal myelination during development^5^. In all cases, a more in-depth knowledge of myelin biology has the potential to provide substantial clinical benefits as no curative treatments currently exist^2^,^3^,^4^,^5^.

Clinically, myelin health is often assessed using magnetic resonance imaging (MRI)^2^,^6^, but the spatial resolution of MRI is not sufficient to visualize the cellular mechanisms of myelination and remyelination (the adult regenerative process). To this end, optical microscopy is more adequate because single cells can be resolved within whole organs^7^,^8^. In addition, longitudinal imaging of biological processes in physiological conditions is enabled by intravital microscopy. Combined with animal models in which fluorescent proteins are expressed in a gene-specific manner, intravital microscopy has been taken advantage of to study oligo-lineage cells, the cells forming myelin sheaths^9^,^10^,^11^,^12^. Unfortunately, a direct assessment of myelin state remains challenging due to the lack of tools to visualize the nanoscopic spatial organization of the several concentric lipid lamellae forming myelin sheaths^13^. Electron microscopy (EM) provides a sufficient spatial resolution^14^, but samples need to be heavily processed and the method cannot be realized *in vivo*.

Here we demonstrate a new system for noninvasive, longitudinal and quantitative optical assessment of the myelin micro- and molecular structure *in vivo*. The system combines a zebrafish spinal cord model together with coherent anti-Stokes Raman scattering (CARS) microscopy and polarization-resolved measurements. We applied this system to the study of developmental myelination and to the screening of promyelination drugs. The ability to observe global and focal changes in the molecular arrangement of myelin is revealed to be essential in order to assess differences that remain elusive at the morphometric level. We believe that this approach will prove valuable for the identification of biomarkers of myelin pathologies and for high-throughput screening of promyelination therapeutic agents.

## Results

### Polarization CARS imaging in zebrafish

**Figure 1:**
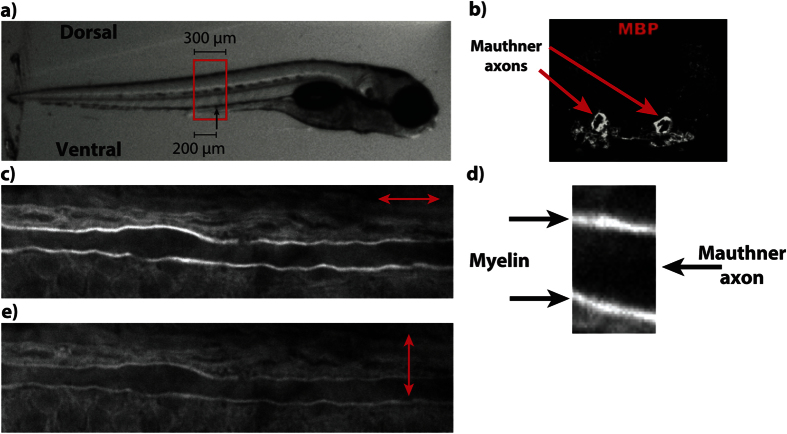
P-CARS zebrafish spinal cord model. (**a**) The same spinal cord segment can be identified on different imaging sessions using morphological references. (**b**) The ventral spinal cord contains two Mauthner axons, one on each lateral side (image width: 80 *μ*m). (**c**) The Mauthner axon can be easily identified with CARS microscopy (image width: 112.5 *μ*m) based on the visualization of (**d**) its myelin sheaths (image width: 7 *μ*m). (**e**) Imaging of the same location as in (**c**) with an orthogonal polarization orientation showing the modulation in CARS intensity. The arrows indicate the orientation of the linear polarization.

We achieved chemically specific imaging of myelin sheaths using CARS microscopy^15^,^16^. CARS uses two short laser pulses at different frequencies to coherently and resonantly excite the vibration of a molecule, which in turn drives the emission of anti-Stokes photons^17^,^18^. The chemical specificity of CARS refers to (1) the selectivity of the molecular vibration probed by the frequency difference between the pump and the Stokes excitation beams and (2) the consequential uniqueness in type of anti-Stokes photon emitter molecules. For myelin imaging, the frequency difference is selected to match the CH_2_ vibration because myelin is lipid-rich. A large myelin-specific contrast is thus obtained in the central nervous system. Myelin imaging in the spinal cord is possible in wild-type zebrafish, but only after a chemical treatment aiming at removing melanophore pigments as they generate a strong background signal (see Methods). The spinal cord can also be optically accessed noninvasively in live nacre zebrafish, a transgenic strain in which melanophore pigments are absent^19^. After defining anatomical locations using morphological references (Fig. 1a), we characterized the morphology of myelinated axons in both wild-type and nacre zebrafish. We found no difference between the two strains by evaluating the axon diameter and the myelin thickness (Supplementary Fig. 1). One feature of the zebrafish spinal cord is the presence of Mauthner axons. The Mauthner axons are larger than other axons and only one is present per lateral side (Fig. 1b). Its identification with CARS microscopy is therefore robust (Fig. 1c,d), making it an ideal model for longitudinal intravital studies where individual axons have to be imaged repeatedly^9^,^10^.

CARS imaging was performed in a polarization-resolved manner (P-CARS). This approach takes advantage of the high level of symmetry in myelin sheaths^15^,^20^,^21^. Indeed, CH_2_ molecules are not randomly distributed within the diffraction limited excitation volume. Their molecular dipoles are partially aligned and this distribution can be characterized by a main orientation and a width. As CARS is a coherent process, the emitted CARS signal intensity will be modulated through phase correlation by the distribution of CH_2_ molecular dipoles and the configuration of the excitation fields polarization^22^. P-CARS consists in the acquisition of multiple CARS images at the same position. Each image is recorded with a different orientation of the collinear polarization of the two excitation beams. As the linear polarizations are rotated^21^, the CARS signal intensity is modulated and this modulation informs on the molecular dipole distribution^23^,^24^,^25^. Figure 1e shows CARS images of a Mauthner axon taken with the orthogonal orientations of the polarization from Fig. 1c to emphasize the modulation in signal intensity. This modulation can be evaluated analytically by performing a fit from which the *myelin modulation* (MM), an index quantifying the myelin subdiffraction spatial structure based on average molecular dipole orientation, is calculated (see Methods and Supplementary information). The MM is independent from the number of Raman scatterers or the sample orientation and takes into account the complete polarization dependence spectrum, i.e the shape of the curve.

### Promyelination drug screening *in vivo*

**Figure 2:**
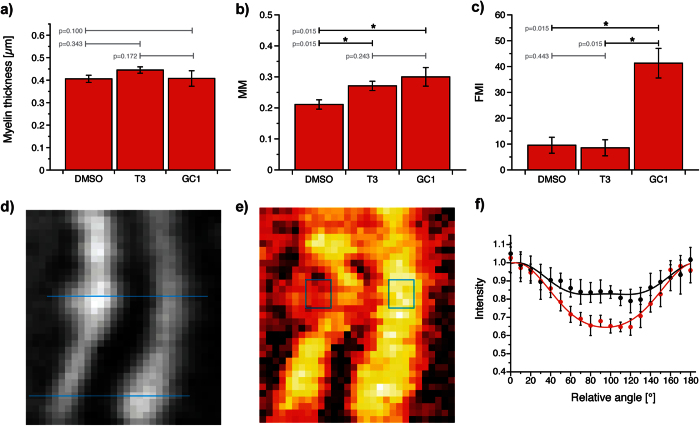
Evaluation of promyelination chemical treatments. (**a**) The myelin thickness, (**b**) the MM, and (**c**) the focal myelination index (FMI) as a function of chemical treatments (Mann-Whitney test, *n* = 4). (**d**) CARS image of a Mauthner axon in a GC1-treated zebrafish. (**d**) Mapping of the MM from P-CARS reveals a discontinuity on the left side. Blue ROIs indicate where the polarization dependence is plot in (**f**). The black curve is from the focal myelin discontinuity (e-left, MM = 0.17 ± 0.04) and the red curve from the normal region (e-right, MM = 0.34 ± 0.03).

We aimed to evaluate the potential for *in vivo* drug screening of P-CARS using the Mauthner myelination zebrafish model. We therefore studied the effects of known promyelination drugs acting on the thyroid hormone pathway: triiodothyronine (T3) and thyroid hormone receptor *β* agonist (GC1)^26^. We chose this model because it is known that both GC1 and T3 treatments promote myelination. It is also known that the GC1 treatment has global deleterious effect on the zebrafish development, while T3 treatment does not result in anatomical malformations when compared to dimethyl sulfoxide (DMSO, control) treated zebrafish. Embryos were imaged after incubation in treated water from 1.5 to 5 days post-fertilization (dpf). The morphological analysis (myelin thickness and axon diameter) did not show differences between T3-, GC1- and the DMSO-treated zebrafish (Fig. 2a and Supplementary Fig. 2). In contrast, P-CARS imaging was able to reveal differential drug effects. First, the MM was significantly larger in both T3 and GC1 chemical treatments, but no differences between them was observed (Fig. 2b). Second, by mapping the MM at every pixels to generate a map of the myelin molecular order, we identified several focal regions where myelin was less ordered in GC1 treated fish (Fig. 2d–f and Supplementary Figs 3 and 4). These events were almost completely absent in DMSO- and T3-treated fish and completely invisible to conventional CARS imaging. The significantly higher focal myelination index (FMI - number of focal regions with lower MM normalized to the analyzed length) in GC1 treated fish is consistent with their general abnormal development (Fig. 2c).

### Intravital imaging of developmental myelination

**Figure 3:**
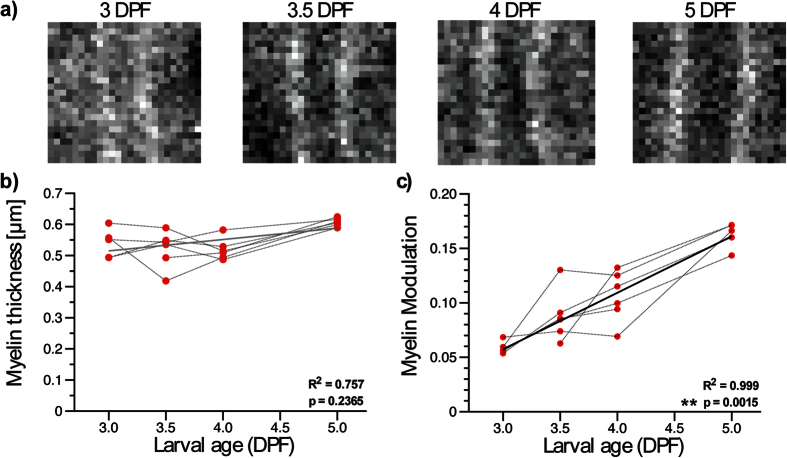
Developmental myelination of the Mauthner axon in live nacre zebrafish. (**a**) CARS images of the Mauthner axon on different days (image width: 8.4 *μ*m). Representative images were selected from longitudinal imaging experiments (repeated imaging over time of same fish). (**b**) The myelin thickness and (**c**) the MM are plotted as a function of larval age. A steady increase in MM, but not in myelin thickness, was measured between 3 and 5 dpf. *R*^2^ for linear regression and p-value for non-zero slope test are given in each graph.

**Figure 4:**
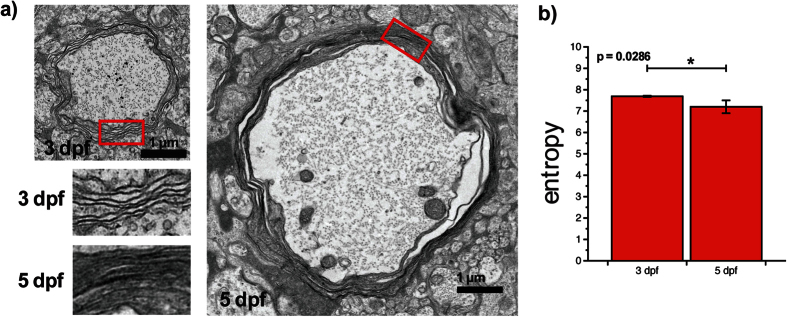
Electron microscopy confirms that P-CARS informs on the nanostructure. (**a**) Electron microscopy images at 3 and 5 dpf show the difference in myelin lamellae organization. (**b**) The entropy decreases significantly between 3 and 5 dpf (Mann-Whitney test, *n* = 4).

The first 5 days after fertilization are a critical period for the myelination of Mauthner axons as no significant morphological changes occur beyond 6 dpf (Supplementary Fig. 5). Imaging at 5 dpf thus informs on the results of myelination. Nevertheless, imaging at multiple time points during this process is necessary to understand the sequence of events regulating it. As a first step in this direction, we demonstrate the noninvasive imaging of myelination in live zebrafish embryos between 3 and 5 dpf (Fig. 3a). Changes in both axon morphology and myelin molecular distribution were measured during this period. At 3 dpf, myelination was partial along the axonal length and in one case completely absent. Imaging earlier time points would therefore not provide additional information. Between 3 and 5 dpf, the axon diameter increased linearly (Supplementary Fig. 6), but the myelin thickness did not significantly change (Fig. 3b). While the myelin thickness remained the same, the average MM on individual axons increased by ∼3 folds (Fig. 3c). This increase translates into the myelin sheath becoming more organized, CH_2_ molecular dipoles becoming more aligned. To further support this observation, EM was performed on 3 and 5 dpf zebrafish and images showing the myelin structure with submicrometer resolution were obtained (Fig. 4a). A significant increase in myelin lamellae organization, as quantified by a decrease in entropy^27^ (see Methods), was observed (Fig. 4b).

## Discussion

We have presented a new approach for the intravital and multi-scale assessment of the myelin structure. The approach consists in combining multiphoton imaging and polarization-resolved measurements to a specific zebrafish spinal cord model. The zebrafish model offers several advantages over mammalian ones. First, zebrafish are sufficiently small for the spinal cord to be imaged noninvasively with multiphoton microscopy during the larval stage. This enables serial imaging of live zebrafish, which only need to be anesthetized. The minimal sample preparation makes zebrafish compatible with automated handling for high-throughput screening^28^. Importantly, their fast *ex utero* development also enables the visualization and assessment of myelination. The scope of the model is not limited to developmental studies. Indeed, myelination often serves as a model of remyelination because of the similitudes between the two processes (recapitulation hypothesis)^29^,^30^,^31^. This is of particular interest as zebrafish react to drugs similarly to humans, making them good candidate for drug screening^32^.

Multiphoton microscopy has become an important tool in molecular and cell biology as it allows biochemically specific imaging in tissue with a subcellular spatial resolution^8^. CARS has mainly been associated with lipid imaging because of the strong signal originating from C-H bond stretches^33^. For instance, CARS has been used to study myelin morphology in the mammalian nervous system, myelin being lipid rich^34^,^35^,^36^. In this work, the myelin microscopic structure was characterized by performing standard morphometric measurements (axon diameter and myelin thickness - see Methods)^36^. All morphological results obtained agreed with previously published EM measurements in fixed and processed samples^37^. The smallest average myelin thickness in our data was of 0.40 *μ*m. This value is below twice the pixel size (225 nm in all images) by 50 nm, but the effective sampling is nonetheless well above the Nyquist criterion. This is true because each measurement was an average of multiple pixels over the axonal length and no localization was performed. The myelin thickness measurement is (spatially) limited by the PSF of the CARS microscope used in this study (0.34 *μ*m or 0.4*λ*_*p*_)^38^. Regardless of these technical considerations, the challenges of morphometrically characterizing myelin very early during development only emphasize the relevance of non-morphological metrics, such as MM and FMI, which are also independent of the PSF of the CARS microscope.

CARS is a label-free technique as it probes single molecular vibrations of endogenous molecules^17^,^18^. The latter fact indicates that polarization-resolved measurements with CARS directly evaluate the average molecular order (CH_2_ molecular dipoles) of the structure of interest within the excitation volume. It is already well known that nonlinear susceptibility tensor elements or equivalent parameters obtained from polarization dependence experiments relate to the degree of molecular alignment^23^,^39^. For CARS, this aspect has been demonstrated in crystal^23^,^40^ and applied to biological samples^21^,^24^,^25^,^41^. As defined here, an increasing MM during development informs on the myelin sheath lamellae becoming more organized, CH_2_ molecular dipoles becoming more aligned. Additional experimental evidence of this fact can be found in Bélanger *et al*.^21^. To further support this observation, we performed EM on 3 and 5 dpf zebrafish and validated the increase in myelin lamellae organization. This provides supplemental evidence to the fact that the MM index informs on the nanoscopic myelin structure.

It is well known and characterized from a decade of work by us and others that CH_2_ dipoles are specifically imaged with CARS in the nervous system at the vibrational frequency probed (2845 cm^−1^)^15^,^16^,^17^,^18^,^36^, and that this detection is polarization dependent. The molecular organization of myelin, with its concentric myelin lamellae, with their concentric phospholipid bilayers, with their long CH_2_ chains is such a periodic and symmetric structure. The known structure, ultra-structure, and molecular structure of myelin have made it possible for several groups to derive expressions modeling very well the polarization dependence in intact myelin^20^,^23^,^24^,^25^. All this symmetry is beautiful and complex.

The MM index and its mapping obtained from P-CARS provided information on the myelin state that could not have been accessible *in vivo* otherwise. Only EM possesses a high enough resolution to resolve individual myelin lamellae but this approach is not compatible with longitudinal studies and cannot be performed in intact tissue^13^,^14^. The P-CARS information is complementary to the morphological one. In both applications illustrated, differences in the myelin structure were observed at the molecular level. In particular, the myelin thickness didn’t change over the observed myelination period, while the MM index had a three-fold increase. For the pro-myelination drug screening, the myelin thickness was the same for all conditions, while a higher order was found in T3- and GC1-treated fish. Additionally, the presence of focal myelin regions with low MM might not affect the global MM value, but could significantly affect myelin function. Therefore, the ability to map the myelin molecular order with a diffraction limited spatial resolution was essential in assessing myelin integrity following drug treatment.

The nature of the altered molecular order in focal region is not known and could not be investigated with EM because of limited sampling along the caudo-rostral axis. Mauthner axons don’t have nodes of Ranvier^42^ at the spinal cord level and are expected to be continuously myelinated in normal conditions. Apparent discontinuities in P-CARS might be due to forming collateral branches. They are unlikely related to myelin sheath junctions between different oligodendrocytes because circum-symmetric discontinuities were not observed. Gaining a better understanding on the origin and nature of these focal regions will be useful in future work to better interpret data.

In this study, sample-induced aberrations and the possible presence of birefringent structures were not evaluated. Zebrafish embryos can be considered relatively transparent (non-scattering) in comparison to the mammalian brain and spinal cord. Low order aberrations are thus the primarily source of signal and resolution loss after focusing through the sample. Such low-order aberrations are not expected to affect the polarization state when focusing through such samples. Establishing the proper experimental models for characterizing possible alterations of polarization states, and then finding novel ways to compensate for them will be the topic of future work. Another caveat is that the Raman spectrum of the sample was not characterized. Possible changes in the spectral signature of myelin from difference in lipid compositions between zebrafish and other mammalian models were assumed to be negligible as the lipid spectral width is very broad around 2845 cm^−1^. This latter fact is well supported by the detailed review on Raman spectroscopy of lipids by Czamara *et al*.^43^. Water signal falling into our anti-Stokes frequency bandwidth, nonresonant contribution, and how they affect P-CARS were also not assessed for this specific model.

In conclusion, we have developed a system to study the structure of myelin sheaths *in vivo* at the microscopic and molecular levels simultaneously. We have demonstrated the system by characterizing developmental myelination in live zebrafish and by assessing the effect of promyelination drugs. Two biomarkers of myelin health (myelin modulation (MM) and number of focal low-MM regions) were introduced and shown to detect subtle difference in the myelin structure. The same biomarkers could potentially be applied to other animal models. It would be of particular interest to investigate the detection of early micro- and nano-lesions in the pathology of multiple sclerosis. It is expected that our approach will become a powerful tool for the identification of new therapeutic avenues for the treatment of demyelinating diseases, especially if combined with high-throughput screening methods^28^.

## Methods

### Optical imaging.

The set up used for CARS imaging is described in detail elsewhere^20^. A schematic of the system is shown in Supplementary Fig. 7. A frequency-doubled Nd:Vanadate mode-locked laser (PicoTrain, High-Q Lasers) pumps an optical parametric oscillator (Levante Emerald, APE-Berlin). The frequency difference of the two excitation fields (816.8 nm and 1064 nm) corresponds to the 2845 cm^−1^ Raman shift of the CH_2_ vibrational resonance^43^. Imaging is performed in an epi-detection configuration with a 60X objective lens (Olympus, UPlanSApo, 1.2 NA, W) or 40X objective lens (Olympus, LUMPlan Fl/IR, 0.8 NA, W). The anti-Stokes CARS signal is separated from the excitation light by a dichroic long-pass filter (Semrock, FF735-Di01-25 × 36) and two laser block filters (Semrock, FF01-750/SP-25). The emission light is further filtered by a band-pass filter (Semrock, FF01-655/40-25) and detected by a photomultiplier tube (Hamamatsu, R3896). We elected the epi CARS configuration for two reasons. First, the use of a single objective lens simplify substantially the complexity of the optical system, which is important in biological applications for robustness. Second, it enables the use of a single CARS and P-CARS imaging platform for both thick/scattering tissue (such as the spinal cord) and transparent tissue often encountered in biological applications. A paired-dichroic (Semrock, FF735-Di01-25 × 36) was positioned near the tube lens to maintain the polarization for all incident linearly polarized fields and a set of wave plates was used to make both excitation fields collinear and a broadband half-wave plate to rotate the orientation of the linearly polarized excitation light^21^. The rotation was automated with a servo motor rotation stage (Newport, SR50CC). The polarization state at the pupil plane was linear as verified by Malus’ Law^21^. Sample-induced aberrations and the possible presence of birefringent structures were not considered. 19 images were recorded at the same position. For each image, the linear polarization of the excitation fields was rotated by 10° 22. The emission light polarization was not analyzed (un-analyzed detection configuration)^44^. The polarization dependence was fitted at every pixels to^20^:


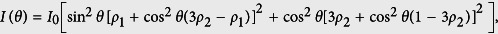


where the terms 
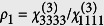
, and 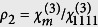 contain the information on the polarization dependence. The myelin modulation (MM) was obtained at every pixel using: 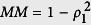 (see Supplementary information).

### Sample preparation and optical imaging procedure

For live zebrafish imaging, embryos were produced by normal matings of nacre; *tg*(*olig2*:EGFP) transgenic zebrafish. Embryos were raised in E3 water [294 mM NaCl, 10 mM KCl, 20 mM CaCl_2_H_2_O, 20 mM MgSO_4_H_2_O] and staged according to days post-fertilization (dpf). Embryos were kept at 28.5 °C except during the 90 minutes imaging sessions (15 min. per embryos) and their transportation between institutes (2 first dpf) where the temperature was of approximatively 24 °C. For imaging, fish were placed laterally on a microscope slides with E3 and 0.02% tricaine anaesthetic. The fish was surrounded by three 150 *μ*m-thick spacer and a coverslip was placed on top. In order to always image the same region of the spinal cord, the yolk sac extension, also generating a large CARS signal due to its lipid content, was used as a morphological reference. For morphometric analysis, two axial-stacks of the spinal cord were taken toward the tail (200 *μ*m) and one toward the head (100 *μ*m). The imaged region was thus always 300 *μ*m long. For P-CARS imaging, a single image series was acquired in the same central region. A total of six zebrafish were imaged (*n* = 6). Between imaging sessions, fish were recovered and put back in E3 water.

For the chemically treated zebrafish, embryos were produced by normal matings of AB wild type fish. Embryos were raised at 28.5 °C in E3 water and staged according to dpf. Stock solutions (10 mM) were made for the following chemicals using concentrated DMSO: triiodothyronine (T3) [Sigma Aldrich], thyroid hormone receptor beta agonist (GC1). The stocks were subsequently diluted initially in DMSO and then in E3 (10 ml) to yield the correct final concentration (1:1000 dilution). Embryos from multiple matings were pooled, bleached and dechorionated at approximately 1 dpf, after which they were distributed into individual 60 mm petri-dishes (34 embryos/dish). At 1.5 dpf the E3 was removed from the dishes and replaced with E3 containing appropriate concentrations of chemicals. Embryos were maintained in the treatments until 5 dpf at which point they were anesthetized with 0.02% tricaine and then fixed overnight with 4% paraformaldehyde at 4 °C. To eliminate melanophore pigments, wild-type zebrafish were bleached with 500 *μ*l of a bleaching solution (8.25 mL DEPC H_2_O, 1 mL 30% H_2_O_2_, 0.5 mL 10% KOH) applied until their eyes begin to turn brown. Fish were washed 2 times for 5 minutes in PBS before a 20% PFA fixation at room temperature. The CARS imaging for morphometric analysis was identical to that of live zebrafish. P-CARS imaging was performed over the same 300 *μ*m region. For each chemical treatment, four zebrafish were imaged (*n* = 4).

All animal experiments were performed in compliance with institutional guidelines and approved by the Institutional Animal Care and Use Committee at University of Colorado. Intravital imaging was performed in compliance with institutional guidelines at Université Laval. Zebrafish embryos are highly sensitive to environmental variables. It is therefore important to avoid comparing different experiments directly and critical to always have a control group or reference time point.

### Electron microscopy

For electron microscopy, 3 and 5 dpf nacre zebrafish embryos were processed by a microwave-assisted protocol. All steps were done in a BioWave Pro microwave (Ted Pella, Inc., Redding, CA). Zebrafish embryos were fixed in a modified Karnovsk’s fixative (2% glutaraldehyde + 2% paraformaldehyde in 0.1 M sodium cacodylate containing 0.025% calcium chloride) as follows: 1 minute 120 Watts (W), 1 minute 0 W, 1 minute 120 W, 10 seconds 650 W, 20 seconds 0 W, 10 seconds 650 W. Specimens were then rinsed twice outside of the microwave for 2.5 minutes each with 0.1 M sodium cacodylate containing 3% sucrose followed by a 40 second 120 W rinse in the BioWave Pro. They were postfixed with 1% osmium tetroxide in water for 1 minute 0 W, 2 minutes 120 W, 2 minutes 0 W, 2 minutes 120 W. The samples were then rinsed briefly with water outside of the microwave, after which they were dehydrated in a graded series of acetone (50, 70, and 90%-once each; 100%-three times) for 40 seconds at 120 W. The remaining infiltration/embedding steps were outside of the microwave. The samples were infiltrated overnight in a 1:1 Epon812:100% acetone mixture, followed by 2 hours in pure epon, and finally embedded in flat embedding molds. Ultrathin sections (70 nm) were collected on 150 copper mesh grids and post-stained with Reynold’s lead citrate and 4% aqueous uranyl acetate for 5 minutes each. Images were acquired with a Gatan UltraScan 1000 2K digital camera on a FEI Technai G2 BioTwin at 80 kV.

### Image processing and analysis

For morphological analysis, individual axons were cropped using ImageJ (NIH, Bethesda, Maryland, USA). For each axon, 12 crops were taken uniformly (at equidistant locations) along the 300 *μ*m imaged region. This systematic random sampling is an unbiased and standard stereological sampling strategy. Each crop extended along the axonal length for increased effective pixel sampling (for at least 4 pixels - Nyquist equivalent criterion of 112.5 nm). Axon diameter and myelin thickness were computed automatically for all crops using an in-house Matlab software (Mathworks, Natick, Massachusetts, USA). Calculation were based on the cumulative integral method^36^,^45^. To evaluate the MM, a region of interest (ROI) was drawn in the myelin sheath and the average MM value was calculated. Electron microscopy images were analyzed by calculating the entropy in at least 12 ROIs at 3 different positions (4 per positions) along the Mauthner axon with Matlab (n = 4), using again a uniform sampling. Only regions with discontinuity in the myelin were excluded. Entropy is a measure of randomness, which is expected to decrease with increasing order. This metric was selected to minimize possible bias compared to manual metrics such as straightness because it is computed automatically. For the developmental myelination results, a linear fit was performed for morphometric parameters and the MM index as a function of the developmental stages. The slope was tested against a non-zero value. A Mann-Whitney test was used for group comparison in the chemical treatments experiment and electron microscopy measurement (*p* < 0.05*, *p* < 0.01** and *p* < 0.001***, Bonferroni correction was applied as needed). All error bars are presented as standard error of the mean.

## Additional Information

**How to cite this article**: Turcotte, R. *et al*. Intravital assessment of myelin molecular order with polarimetric multiphoton microscopy. *Sci. Rep.* **6**, 31685; doi: 10.1038/srep31685 (2016).

## Supplementary Material

Supplementary Information
