# Supplementary material for: Intravital assessment of myelin molecular order with polarimetric multiphoton microscopy
Source: Sci Rep. 2016 Aug 19;6:31685. doi: 10.1038/srep31685 (PMC4990840; doi:10.1038/srep31685)
Supplement: Supplementary Information [file srep31685-s1.pdf]

SUPPLEMENTARY INFORMATION

Intravital assessment of myelin molecular order with polarimetric multiphoton microscopy

Raphaël Turcotte,[1,+] Danette J. Rutledge,[2,+] Erik Bélanger,[1] Dorothy Dill,[2] Wendy B. Macklin,[2] and Daniel C. Côté.[1,3,*]

[1] Centre de recherche de l'Institut universitaire en santé mentale de Québec, Université Laval, Québec, Qc G1J 2G3, Canada [2] Department of Cell and Developmental Biology, School of Medicine, University of Colorado Anschutz Medical Campus, Aurora, CO 80045, USA [3] Centre d'Optique, Photonique et Laser, Université Laval, Québec, QC G1V 0A6, Canada [+] These authors contributed equally to this work.

*Correspondence: daniel.cote@crulrg.ulaval.ca

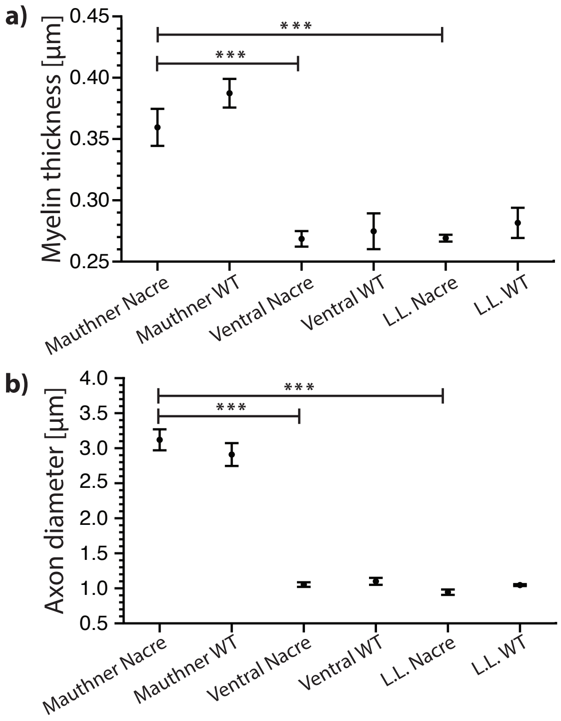

Supplementary Figure 1. Morphometric comparison between wild-type zebrafish and nacre mutant. At least 90 axon segments were analyzed per fish in the lateral line (L.L.) and the ventral spinal cord. Mauthner axons were analyzed as described in the methods. (a) Myelin thickness and (b) axon diameter vs zebrafish type and white matter region. An one-way ANOVA test revealed no difference between the wild-type and nacre mutant. Differences were only observed between white matter regions (One-way Anova test, $n = 4$).

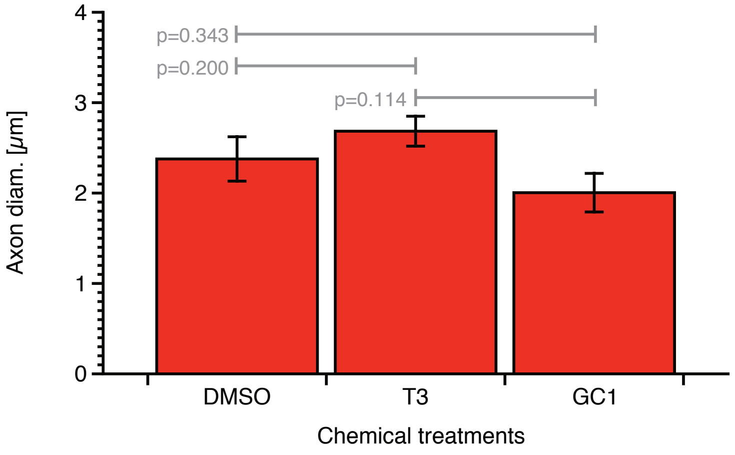

Supplementary Figure 2. Morphological analysis of the Mauthner axon in T3-treated, GC1-treated and DMSO control zebrafish. The axon diameter was not different between chemical treatments and the control (Mann-Whitney test, n=4).

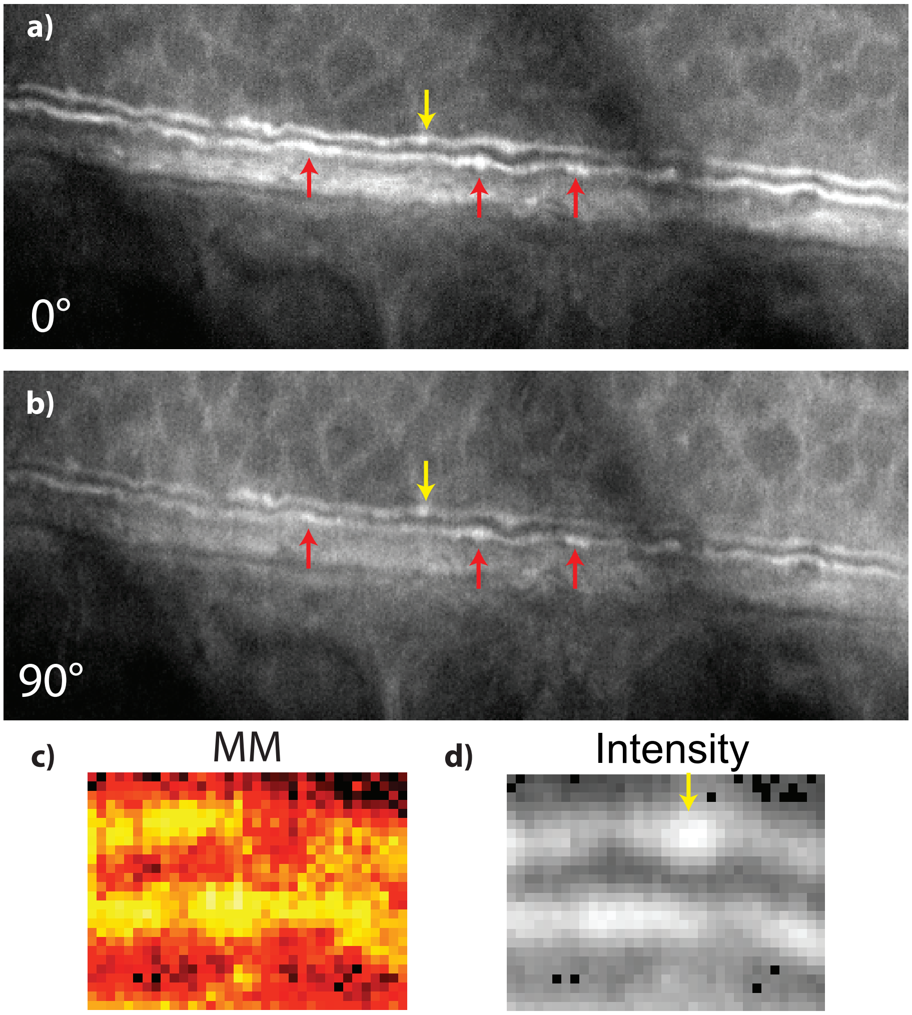

Supplementary Figure 3. CARS images of a 5 dpf GC1-treated zebrafish taken at two orthogonal polarization orientations: 0° and 90° (width: 112.5 μm). Arrows point MM discontinuities that have a smaller intensity modulation. Comparing MM and intensity images (width: 8.5 μm) from the yellow arrow, the discontinuities can easily be discerned when looking at the discontinuity in the MM image.

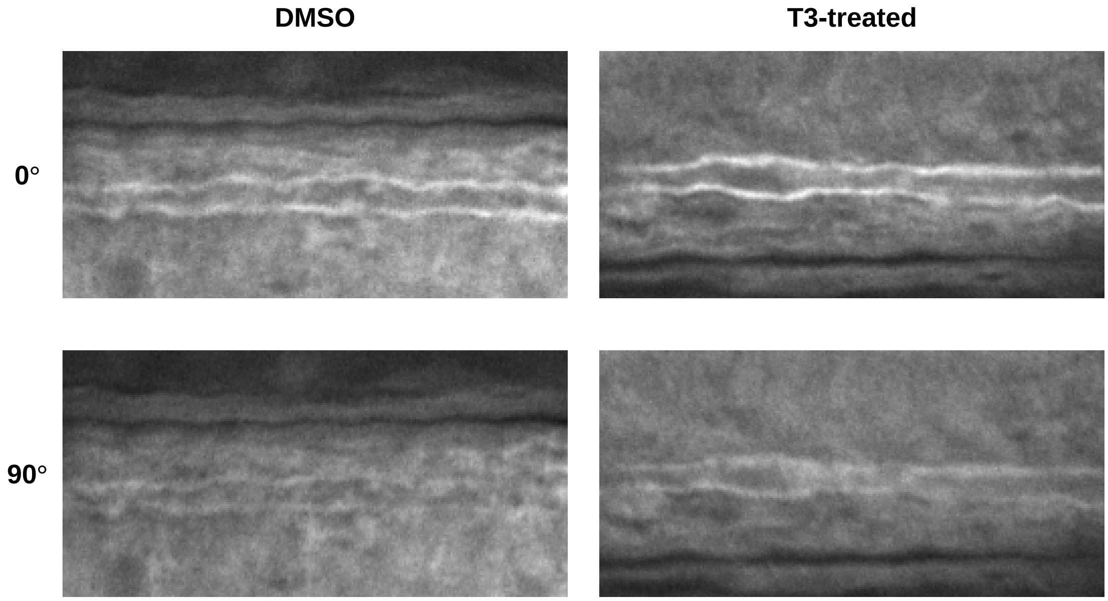

Supplementary Figure 4. CARS images of a 5 dpf DMSO and T3-treated zebrafish taken at two orthogonal polarization orientations: 0° and 90° (width: 56.25 μm).

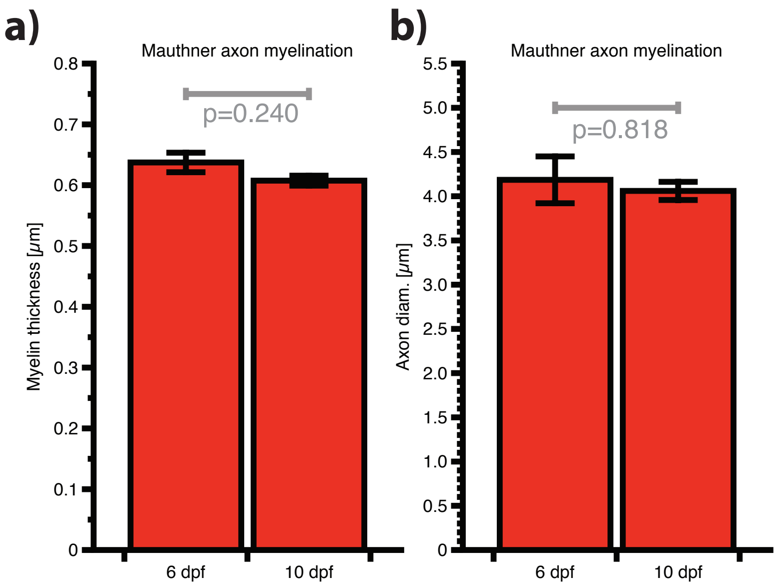

Supplementary Figure 5. Mauthner axon development in fixed nacre fish. (a) Myelin thickness and (b) axon diameter as a function of larval age. No difference were observed for all parameters between 6 and 10 dpf (Mann-Whitney test, $n = 6$).

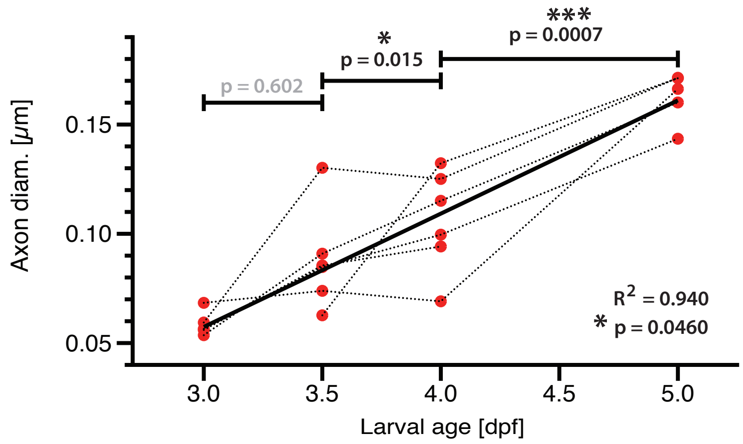

Supplementary Figure 6. Axonal growth of Mauthner axons during development in live nacre zebrafish. A steady increase in axon diameter was measured between 3 and 5 dpf. R^2 for linear regression and p-value for non-zero slope test are given in each graph. (n=6 for all ages, except at 3 dpf where n=5).

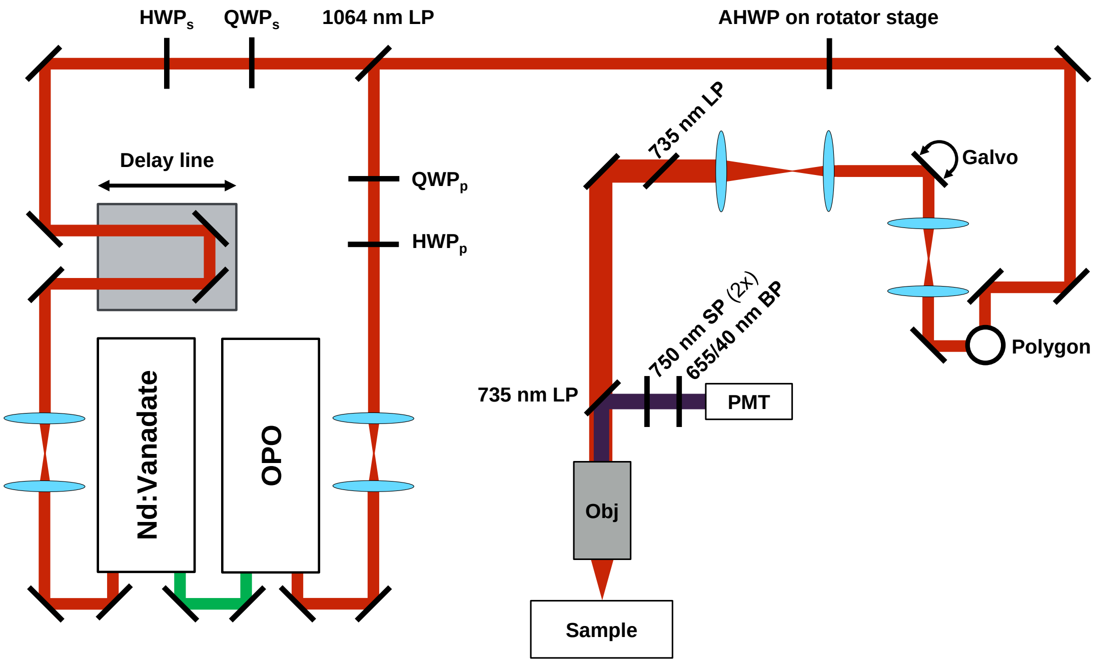

HWP$_s$: AO15Z 1/2 1064, HWP$_p$: AO15Z 1/2 817, QWP$_s$: AO15Z 1/4 1064, QWP$_p$: AO15Z 1/4 817
From Tower Optical Corporation

Supplementary Figure 7. Schematic of the optical scanning microscope for CARS and P-CARS imaging indicating the position of polarization-control elements.

Derivation of the myelin modulation index

The CARS signal polarization dependence for pump and Stokes field with collinear polarization can be expressed as (Bélanger *et al.*, 2009):

$$
\begin{aligned}
I(\theta) \propto \quad & E_p^4 E_s^2 \Big[\sin(\theta)^2 [\chi^{(3)}_{3333} + \cos(\theta)^2 [3\chi^{(3)}_m - \chi^{(3)}_{3333}]]^2 \\
& + \cos(\theta)^2 [3\chi^{(3)}_m + \cos(\theta)^2 [\chi^{(3)}_{1111} - 3\chi^{(3)}_m]]^2\Big],
\end{aligned}
\tag{1}
$$

where θ is the incident polarization orientation, E_p and E_s are the pump and stokes fields, and $\chi^{(3)}_{3333}$, $\chi^{(3)}_{1111}$, and $\chi^{(3)}_m$ are the three non-zero independent nonlinear susceptibility tensor elements probed. The extrema of Eq. 1 can be found by taking its derivative:

$$
\begin{aligned}
dI(\theta)/d\theta \propto\ & 2(\chi^{(3)}_{3333} + \cos(\theta)^2(3\chi^{(3)}_m - \chi^{(3)}_{3333}))^2 \sin(\theta)\cos(\theta) \\
& - 4(\chi^{(3)}_{1111} - 3\chi^{(3)}_m)\cos(\theta)^3 \sin(\theta)(3\chi^{(3)}_m + (\chi^{(3)}_{1111} - 3\chi^{(3)}_m)\cos(\theta)^2) \\
& - 2\cos(\theta)\sin(\theta)(3\chi^{(3)}_m + (\chi^{(3)}_{1111} - 3\chi^{(3)}_m)\cos(\theta)^2)^2 \\
& - 4(3\chi^{(3)}_m - \chi^{(3)}_{3333})\cos(\theta)\sin(\theta)^2(\chi^{(3)}_{3333} + (3\chi^{(3)}_m - \chi^{(3)}_{3333})\cos(\theta)^2),
\end{aligned}
\tag{2}
$$

and then finding the zeros. For $\theta \in [0, \pi[$, Eq. 2 takes a value of zero for $\theta = 0$ and $\pi/2$:

$$
I(\theta = 0)_{max} = (\chi^{(3)}_{1111})^2 \text{ and } I(\theta = \pi/2)_{min} = (\chi^{(3)}_{3333})^2. \tag{3}
$$

The myelin modulation (MM) is defined as:

$$
MM \equiv \frac{I_{max} - I_{min}}{I_{max}} = \frac{(\chi^{(3)}_{1111})^2 - (\chi^{(3)}_{3333})^2}{(\chi^{(3)}_{1111})^2} = 1 - (\chi^{(3)}_{3333}/\chi^{(3)}_{1111})^2. \tag{4}
$$
